# Maternal point‐of‐care ultrasound training during Maternal Fetal Medicine fellowship: A national survey

**DOI:** 10.1002/pmf2.70348

**Published:** 2026-06-22

**Authors:** Elsa Parra, Sheree Carter, Anna Joy Rogers, Kerri Brackney

**Affiliations:** ^1^ Division of Maternal Fetal Medicine University of Tennessee Health Science Center Memphis Tennessee USA; ^2^ Division of Obstetrics & Gynecology University of Tennessee Health Science Center Memphis Tennessee USA

**Keywords:** maternal POCUS, MFM, obstetrics, point‐of‐care ultrasound

## Abstract

**Introduction:**

Point‐of‐care ultrasound (POCUS) is widely used to facilitate management of critical patients. Literature about maternal POCUS (M‐POCUS) is limited, but there is interest in its potential to optimize care for high‐risk obstetric patients. Currently, there is no data on the frequency of M‐POCUS use among Maternal Fetal Medicine (MFM) fellows in training. We aimed to determine what proportion of MFM fellows perform M‐POCUS, and whether exposure to an obstetric intensive care unit (OBICU) impacts M‐POCUS utilization.

**Methods:**

This was a cross‐sectional survey of fellows in the United States. An anonymous 20‐item web‐based survey was designed to ascertain fellow exposure and interest in M‐POCUS training. We defined M‐POCUS as maternal cardiac, lung, and/or abdominal ultrasound, and excluded feto‐placental assessment. We directly contacted 382 fellows and 112 program coordinators and directors via email. Surveys were distributed by email twice weekly and by text message weekly in March, 2025. The analysis was descriptive, with proportions and medians used when appropriate. Comparisons of participant demographics and responses were performed with a chi‐square or Fisher's exact test when appropriate, and *p* < 0.05 was considered significant. Univariate regression was used to perform odds ratios (ORs) with 95% confidence intervals (CIs).

**Results:**

The response rate was 56.3% (*n* = 215/382). MFM fellows with OBICU exposure did not report a significant difference in M‐POCUS use (*p* = 0.18). Overall, 60.0% of respondents performed M‐POCUS at least once during fellowship. The highest predictors of performing an M‐POCUS exam were self‐reported adequate M‐POCUS training (OR, 19.29; 95% CI, 5.79–64.32) and observing an M‐POCUS exam during MFM fellowship (OR, 9.44; 95% CI, 4.70–18.98). Almost all respondents (96.7%, *n* = 208/215) are interested in pursuing further M‐POCUS training.

**Conclusion:**

Although OBICU exposure during MFM fellowship did not alter M‐POCUS use among respondents, they generally perceive value in this skill and have interest in further training opportunities.

## Graphical abstract

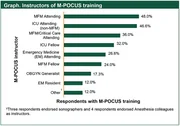

## 1 INTRODUCTION

Point‐of‐care ultrasound (POCUS) is a targeted, real‐time, bedside assessment that can decrease cost, time delay, and radiation exposure for ill patients [1, 2]. Its value has been broadly accepted for bedside assessment in acute care settings [3, 4]. The Accreditation Council of Graduate Medical Education (ACGME) now cites POCUS skills as core competencies for trainees in Emergency Medicine, Anesthesiology, Pulmonary Disease, and Critical Care Medicine [5, 6, 7, 8].

Obstetric POCUS is often limited to feto‐placental evaluation despite the ubiquitous presence of ultrasound for diagnostic purposes in obstetrics [2, 9]. Maternal POCUS (M‐POCUS) proposes to focus on organs of the pregnant patient such as the lungs, heart, inferior vena cava, and abdomen to guide diagnosis and therapeutic interventions in complex obstetric settings [2, 3, 4, 10, 11, 12, 13]. There are limited studies that directly evaluate M‐POCUS [3]. Recent literature calls for the implementation of M‐POCUS as a means of improving care of the pregnant patient [1, 2, 3, 12, 14].

The aim of this study was to assess how often Maternal Fetal Medicine (MFM) fellows perform M‐POCUS to facilitate clinical management. Based on the prevalence of POCUS in critical care settings, we hypothesized that MFM fellows with self‐reported access to an obstetric intensive care unit (OBICU) would perform M‐POCUS exams more frequently.

## 2 METHODS

This was a cross‐sectional survey conducted in March 2025 of all enrolled MFM fellows in the United States. The online survey was conducted in compliance with the Checklist for Reporting Results of Internet E‐Surveys (CHERRIES) guidelines. Institutional Review Board approval was obtained for all study materials and procedures.

### 2.1 Target population

The Society for Maternal Fetal Medicine (SMFM) fellowship directory was used to generate a registry of fellows eligible for survey participation. This registry was supplemented by information from program coordinators, drawn from institutional websites, or published in societal conference materials. Fellows were excluded if they were on extended leave during the survey period, were dismissed from programs, had non‐functioning emails, or did not complete the survey. The final contact list contained 394 fellows and 112 program coordinators or program directors representing 106 of 107 programs based on National Resident Matching Program data for MFM fellowship match years 2022–2024.

### 2.2 Recruitment strategy

Emails to fellows and program coordinators or directors were sent by the primary investigator twice weekly during the month of March. Emails described the aims of the project and included a link to access the survey. Program coordinators or directors were additionally asked to forward the survey link via email to their current fellows. To improve participation, additional survey invitations were distributed weekly in a WhatsApp text to each fellowship class. Survey completion was incentivized by offering optional entry into a drawing for one of five $20 Amazon gift cards. Interested participants submitted a contact email at the end of the survey through an anonymized link. Browser cookies were assigned to the initial survey page to prevent duplicated submissions, and internet protocol addresses were blocked to maintain anonymity.

### 2.3 Survey development and administration

The 20‐item exploratory survey containing four conditional questions was developed by two MFM physician authors. A survey of POCUS training among Nephrology fellows was used as a framework [15]. The survey was distributed using Qualtrics software over eight screen pages and with an estimated duration of less than 10 min. Four independent reviewers, including MFM physicians and research coordinators, were asked to iteratively pilot and refine the survey until a final version was reached.

In the survey, POCUS was explicitly defined as “a noninvasive, bedside extension to the physical exam which facilitates immediate assessment of conditions which benefit from timely diagnosis” [3]. Additionally, M‐POCUS was defined as “maternal assessment (e.g. fluid status, cardiac strain, intraabdominal hemorrhage) and does not include feto‐placental assessment.” Participants were required to acknowledge these definitions prior to advancing in the survey. Survey questions covered participant demographics, frequency of M‐POCUS use, M‐POCUS training methods and instructors, perceived relevance of M‐POCUS, and interest in obtaining further M‐POCUS training (Appendix A in the Supporting Information).

### 2.4 Data analysis

The objective of the study was to determine the frequency of M‐POCUS use clinically and the potential impact of self‐reported OBICU exposure in MFM fellowship on M‐POCUS frequency. We also aimed to determine M‐POCUS training characteristics and fellows’ interest in this skill.

Descriptive statistics were used to report the demographic data, recruitment rate, completion rate, survey duration, and exposure to and interest in M‐POCUS training. An enthusiasm composite was created to include those who stated interest in obtaining M‐POCUS training, those who agreed that M‐POCUS would enhance clinical abilities, and those who desired any mode of future training. The composite was scaled from 0 to 1 and then dichotomized such that composite scores > 0.5 were labeled “enthusiastic.” For comparative analysis, race and ethnicity, and M‐POCUS instructor categories were condensed.

Pearson's chi‐square test and Fisher's exact tests were used as appropriate to compare proportions. An alpha criterion of 0.05 was used to determine statistical significance. Univariate logistic regression was used to estimate unadjusted odds ratios (ORs) with 95% confidence intervals (CIs) for the association between each characteristic and M‐POCUS performance. The recruitment rate was determined by dividing the number of respondents who acknowledged informed consent by the total number of individuals who had access to the survey during the study period. The response rate was determined by dividing the number of participants who completed the survey by the number of individuals asked to participate. The completion rate was calculated by dividing the number of those who completed the last survey question by the number of participants who consented to the survey. All analyses were conducted with SAS 9.4 software.

## 3 RESULTS

**FIGURE 1:**
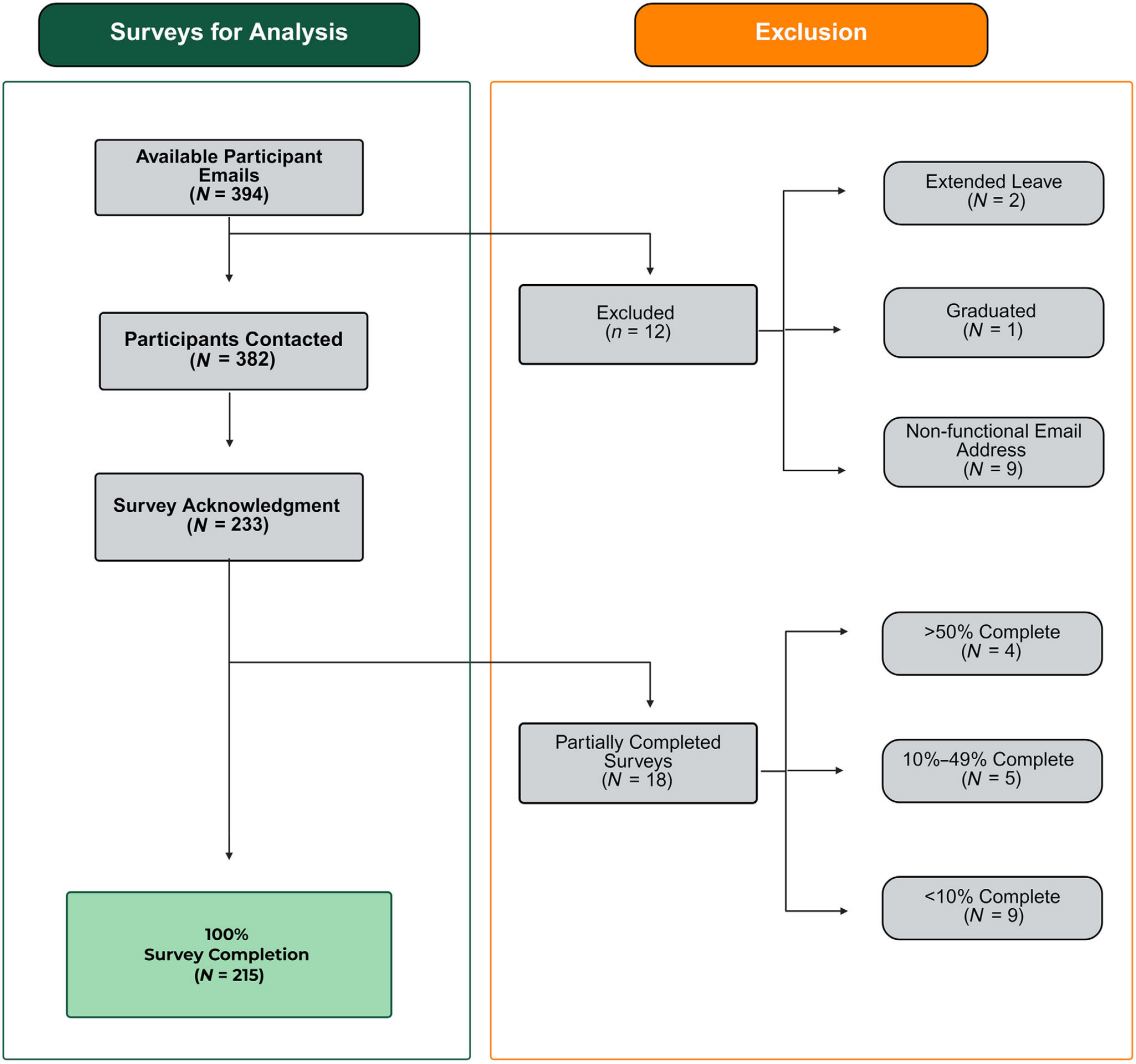
Flow diagram of recruitment and analysis. Created in BioRender (Carter 2026; https://BioRender.com/sbfi1q2) [16].

Out of 394 fellows included in the directory, 12 were excluded from recruitment and response rate assessments (Figure 1). The recruitment rate was 61% (*n* = 233/382), the response rate was 56.3% (*n* = 215/382), and the completion rate was 92.3% (*n* = 215/233). The median duration for survey completion was 2.2 minutes (IQR, 1.7–3.2 min).

Respondent demographic data can be found in Table 1. Approximately one third of all responses were attributed to each fellowship class. Most respondents identified as non‐Hispanic White women and attended fellowship in the Northeast or South regions. Most respondents have observed an M‐POCUS exam in MFM fellowship, and most have performed an M‐POCUS exam for clinical management during MFM fellowship. Overall, 17.7% perform ≥ 1 M‐POCUS exam per month, 42.3% perform < 1 M‐POCUS exam per month, and 40.0% have never performed an M‐POCUS exam. Only 19.5% of respondents reported dedicated MFM fellowship training in an OBICU.

**TABLE 1:** Survey respondent characteristics.^a^

Characteristics	*n* (%)
Race and ethnicity	
White non‐Hispanic	135 (62.8)
Asian non‐Hispanic	32 (14.5)
Hispanic or Latina/o	18 (18.4)
Black or African American non‐Hispanic	14 (6.5)
Other/Mixed	10 (4.6)
Prefer not to disclose	6 (2.8)
Gender identity	
Woman	172 (80.0)
Man	39 (18.1)
Prefer not to disclose	4 (2.9)
Fellowship program region^b^	
South	68 (31.6)
Northeast	67 (31.2)
West	41 (19.1)
Midwest	39 (18.1)
Fellowship program year	
PGY5	71 (33.0)
PGY6	77 (35.8)
PGY7	67 (31.2)
Observed M‐POCUS in MFM fellowship	
No	60 (27.9)
Yes	155 (72.1)
Have performed M‐POCUS exam	
No	86 (40.0)
Yes	129 (60.0)
Have an OBICU in MFM fellowship	
No	173 (80.5)
Yes	42 (19.5)
M‐POCUS exam frequency	
Never	86 (40.0)
<1 exam per month	91 (42.3)
≥1 exam per month	38 (17.7)

^a^ *n* = 215.

^b^ Regions listed in Appendix Bin the Supporting Information.

Respondents were asked how strongly they agreed or disagreed with the following statement: “I received adequate instruction to independently perform M‐POCUS during MFM fellowship.” Of the 215 respondents, those who “strongly” or “somewhat” agreed represented 26.0%. Only 34.9% of respondents have received any M‐POCUS training in their career. Most participants “strongly” and “somewhat” agreed (64.7% and 30.7% respectively) that M‐POCUS training would enhance their clinical ability. Almost all participants are interested in further M‐POCUS training and would pursue either an “SMFM Fellow Lecture or online module” or a “hands‐on course” at a national or international conference (77.7% and 19.0%, respectively). There were 195 (90.7%) respondents who qualified as “enthusiastic” in the composite score.

Table 2 shows the characteristics of respondents who received M‐POCUS training. Most respondents acquired M‐POCUS training during MFM fellowship. Those whose M‐POCUS training occurred predominately outside of MFM fellowship underwent training mostly or exclusively in residency for nearly all respondents. Respondents were asked to select any category of instructor and any training strategy utilized in their M‐POCUS training (Graph). MFM attendings were the most common instructors of the individual instructor categories. After grouping, M‐POCUS instructors were mostly MFM physicians, which included MFM attendings, MFM and Critical Care attendings, and MFM fellows. The most common training strategy was “guided scanning with an instructor.” OBICU did not have a significant impact on M‐POCUS use (*p* = 0.18) (Table 3).

**TABLE 2:** Characteristics of respondents trained in M‐POCUS.^a^

Characteristics	*n* (%)
Timing of M‐POCUS training	
During MFM fellowship	58 (77.3)
Not during MFM fellowship^b^	
Residency	16 (21.3)
Medical school	4 (5.3)
Other (military, courses, other fellowship)	3 (4.0)
M‐POCUS instructors^b^, ^c^	
Any MFM physicians	52 (69.3)
Any ICU physicians	42 (56.0)
Any EM physicians	24 (32.0)
Other^d^	21 (28.0)
M‐POCUS training strategies^b^	
Guided scanning with instructor	58 (77.3)
Independent scanning	47 (62.6)
Lectures	41 (54.6)
POCUS conference or workshop	22 (29.3)
Simulated session with mannequin	19 (25.3)
Online module	11 (14.6)

^a^ *n* = 75.

^b^ Not mutually exclusive characteristics.

^c^ Any Maternal Fetal Medicine (MFM) physician includes: MFM attending, MFM/Critical Care attending, MFM fellow. Any intensive care unit (ICU) physician includes ICU attending and fellow. Any Emergency Medicine (EM) physician includes EM attending and resident.

^d^ Includes generalist obstetricians, sonographers, anesthesiologists, and radiologists.

**TABLE 3:** Respondent characteristics associated with M‐POCUS performance.

Characteristics	OR (95% CI)	*p* value
Race and ethinicity		0.46^a^
White non‐Hispanic	Ref	
Asian non‐Hispanic	1.02 (0.46, 2.26)	
Black or African American non‐Hispanic	1.70 (0.56, 5.13)	
Hispanic/other/mixed	1.70 (0.80, 3.63)	
Gender identity		0.39^a^
Man	Ref	
Woman	0.62 (0.30,1.30)	
Prefer not to disclose	0.44 (0.03, 7.72)	
Fellowship program region^c^		0.48^a^
Midwest	Ref	
Northeast	1.30 (0.58, 2.90)	
South	0.92 (0.42, 2.04)	
West	1.66 (0.67, 4.15)	
Fellowship program year		0.14^a^
PGY5	Ref	
PGY6	1.36 (0.71, 2.62)	
PGY7	2.01 (1.00, 4.03)	
Have received M‐POCUS training	6.71 (3.26, 13.80)	<0.0001^a^
Have adequate M‐POCUS training	19.29 (5.79, 64.32)	<0.0001^b^
Observed M‐POCUS in MFM fellowship	9.44 (4.70, 18.98)	<0.0001^a^
Have an OBICU in MFM fellowship	1.63 (0.79, 3.34)	0.18^a^
Interest in further training		0.32^a^
Hands‐on course	Ref	
SMFM fellow lecture or online module	0.66 (0.32, 1.37)	
Not interested	0.35 (0.07, 1.79)	
Enthusiasm for M‐POCUS	1.52 (0.30, 7.70)	0.34^a^

^a^ Chi‐square test; Alpha = 0.05.

^b^ Fisher's exact test; Alpha = 0.05.

^c^ List of regions in Appendix B.

Respondent characteristics associated with performing M‐POCUS are listed in Table 3. There was a significant difference in M‐POCUS frequency among respondents who received any M‐POCUS training (*p* < 0.0001), those with self‐reported adequate M‐POCUS training (*p* < 0.0001), and those who observed M‐POCUS during MFM fellowship (*p* < 0.0001). Respondents with “adequate” M‐POCUS training had 19.29 times increased odds of clinically applying M‐POCUS (95% CI, 5.79–64.32). Respondents with any M‐POCUS training as well as those who observed M‐POCUS during MFM fellowship also had higher odds of using M‐POCUS (OR, 6.71; 95% CI, 3.26–13.80 and OR, 9.44; 95% CI, 4.70–18.98).

Table 4 demonstrates characteristics of respondents trained in M‐POCUS that are associated with M‐POCUS use. There was a significant difference in M‐POCUS frequency among respondents with M‐POCUS training from an MFM or ICU (non‐MFM) physician, and those who used “guided scanning with an instructor” or “independent scanning” as a training strategy (*p* = 0.01, *p* = 0.03, *p* = 0.01, and *p* = 0.01 respectively). Respondents with any MFM physician as an instructor had the highest odds of performing M‐POCUS (OR, 5.25; 95% CI, 1.36–20.30) among instructors, while “independent scanning” was the training strategy most predictive of M‐POCUS use (OR, 5.87; 95% CI, 1.41–24.47).

**TABLE 4:** Trained respondent characteristics associated with M‐POCUS performance.

Characteristics	OR (95% CI)	*p* value^a^
M‐POCUS Instructor^b^, ^c^		
Any MFM physician	5.25 (1.36, 20.30)	0.01
Any ICU physician	4.16 (1.01, 17.19)	0.03
Any EM physician	0.80 (0.21, 3.03)	0.25
Training modality^b^		
Guided scanning with instructor	5.78 (1.50, 22.37)	0.01
Independent scanning	5.87 (1.41, 24.47)	0.01
Lectures	0.65 (0.17, 2.43)	0.21
POCUS conference or workshop	1.13 (0.27, 4.71)	0.28
Simulated session with mannequin	0.54 (0.14, 2.08)	0.18

^a^ Fisher's exact test; alpha = 0.05.

^b^ Categories are not mutually exclusive.

^c^ Any Maternal Fetal Medicine (MFM) physician includes: MFM attending, MFM/Critical Care attending, MFM fellow. Any intensive care unit (ICU) physician includes ICU attending and fellow. Any Emergency Medicine (EM) physician includes EM attending and resident.

Participants were asked to rate the importance of the following POCUS applications in MFM training: “abdominal focused assessment with sonography for trauma (FAST),” “cardiac assessment,” “inferior vena cava (IVC) assessment,” “lung assessment,” or “other.” Most of the 215 respondents felt that abdominal FAST (89.3%), cardiac assessment (81.9%), IVC assessment (72.6%), or lung assessment (66.5%) were either “somewhat important” or “very important.”

## 4 DISCUSSION

This national survey captured the M‐POCUS experience among a majority of US MFM fellows (56.3%). Most respondents (60.0%) have performed an M‐POCUS exam for clinical management during MFM fellowship even though only a minority have received any M‐POCUS training (34.9%) or felt they had adequate M‐POCUS training (26.0%). The highest predictors of performing an M‐POCUS exam were self‐reported adequate M‐POCUS training (OR, 19.29; 95% CI, 5.79–64.32) and observing an M‐POCUS exam during MFM fellowship (OR, 9.44; 95% CI, 4.70–18.98). Although OBICU exposure during MFM fellowship was not associated with performing M‐POCUS (*p* = 0.18), there was an association among those with M‐POCUS training from an ICU physician (OR, 4.16; 95% CI, 1.01–17.19). This finding suggests that accessibility to a willing instructor during fellowship may be more indicative of M‐POCUS use than merely having a critical care environment dedicated to pregnant patients.

Recent literature recommends incorporating M‐POCUS into the obstetric toolkit because of its potential to improve care in acute settings and thus help mitigate the high US maternal mortality rate [2, 3, 17]. A cross‐sectional review of ICU settings demonstrated that POCUS use helped confirm or change a diagnosis in 84% of cases and facilitated a change in treatment, imaging, or triage for 69% of cases [18]. The clinical value of POCUS application has translated into a demand for this training that starts in medical school. Surveys from 2021 and 2025 demonstrate that over 60% of US medical schools now have a POCUS curriculum to accommodate responsible incorporation of POCUS into medicine [19, 20]. Over 90% of survey respondents seem to echo this sentiment and felt M‐POCUS training would enhance their clinical acumen with 19.0% even willing to travel to national and international conferences for hands‐on courses.

MFM physicians are uniquely positioned to adopt an M‐POCUS skillset based on the prevalence of ultrasound in the specialty. Emergency Medicine has a well‐defined curriculum for POCUS training. In Emergency Medicine curricula, 25–50 quality‐reviewed exams are required per application to demonstrate proficiency as subsequent POCUS applications employ the same foundational skills [21]. This concept may be demonstrated in this survey as merely observing M‐POCUS during MFM fellowship is a great predictor of M‐POCUS use in fellowship (OR, 9.44; 95% CI, 4.70–18.98). Additionally, self‐reported adequate M‐POCUS training and observing M‐POCUS during fellowship were the highest predictors of M‐POCUS use, which suggests MFM physicians may be able to readily apply and become proficient in M‐POCUS because of the ultrasound skill required by the specialty. These findings also may be the result of incorporating an ICU rotation for MFM certification in response to the “Putting the ‘M’ back in Maternal‐Fetal Medicine” session [22] POCUS is a core competency of Critical Care Medicine and MFM fellows may have observed or practiced POCUS during ICU rotations and applied these skills in obstetric patients. Lastly, respondents had the highest odds of performing M‐POCUS if at least one instructor was an MFM physician compared to other instructors (OR, 5.25; 95% CI, 1.36–20.30). This finding may be due to an institutional disposition toward M‐POCUS; however, it may also indicate that MFM trainees and attendings can have a significant impact on increasing the application of M‐POCUS for critical obstetric patients.

This study is the first known national survey assessing M‐POCUS use and training among MFM fellows. There are no surveys of M‐POCUS use or training even among US Obstetrics and Gynecology (OBGYN) residents. This study represents a gap in the literature as we found that nearly all trainees had M‐POCUS training in residency if training occurred outside of MFM fellowship; however, respondents with M‐POCUS training in fellowship were not asked if they also received any M‐POCUS training in residency. Further studies could be used to determine the content and prevalence of M‐POCUS curricula in OBGYN residency. For example, there are various applications of POCUS to assess different organ systems. The abdominal focused assessment with sonography for trauma (FAST) exam is one application of POCUS commonly used to assess intra‐abdominal hemorrhage in trauma or postoperative patients. This application may or may not be more readily taught in OBGYN residency compared to cardiac POCUS applications, which assist in evaluating heart failure. Follow‐up surveys can determine which M‐POCUS applications are taught in residency, which instructors are providing training, and how many OBGYN residents are utilizing these skills.

Another strength is the high response and completion rates of this study. The median duration to complete the survey was approximately 2 min, which likely contributed to the high completion rate (92.3%). Lastly, the survey was completed over the course of a month, which improves internal validity as there is limited time for the cohort's skillset to change during this period. This survey was naturally limited by selection bias. Additionally, no definition was provided for what constitutes an OBICU; thus, exposure to an OBICU may be inaccurately reported. Furthermore, validated questions were not available for this survey. Both attributes can result in a response bias.

## 5 CONCLUSION

There is an overwhelming interest in cultivating M‐POCUS skills to optimize care for critically ill obstetric patients even beyond the intensive care unit. Many MFM providers, including fellows, already employ these skills to supplement bedside evaluations. Further studies can determine the content and prevalence of POCUS curricula in Obstetrics and Gynecology residency as well as MFM fellowship.

## AUTHOR CONTRIBUTIONS

**Sheree Carter**: Visualization; project administration; resources; data curation. **Elsa Parra**: Conceptualization; investigation; funding acquisition; writing—original draft; methodology; validation; visualization; writing—review and editing; formal analysis; project administration; data curation; supervision; resources. **Anna Joy Rogers**: Conceptualization; formal analysis; visualization; data curation; writing—review and editing. **Kerri Brackney**: Conceptualization; methodology; validation; visualization; writing—review and editing; project administration; supervision.

## CONFLICT OF INTEREST STATEMENT

The authors declare no conflicts of interest.

## Supporting information



Supporting information

## Data Availability

The data that support the findings of this study are available from the corresponding author upon reasonable request.
