# Supplementary material for: Maternal point‐of‐care ultrasound training during Maternal Fetal Medicine fellowship: A national survey
Source: Pregnancy (Hoboken). 2026 Jun 22;2(4):e70348. doi: 10.1002/pmf2.70348 (PMC13344261; doi:10.1002/pmf2.70348)
Supplement: Supplementary file 1 [file PMF2-2-e70348-s001.docx]

Appendix

Survey Questions

The first page of the survey reviewed the aims of the project, estimated time to complete the survey, contact information of the primary investigators, notice of survey completion incentive, and confirmation of anonymity. Participants were required to acknowledge this statement and thus complete the informed consent process to initiate the survey. Participants were able to navigate forward and back between pages and change answers as desired. Participants could not exit and return to the survey. Some questions, specifically those tied to the primary outcome, required a response before participants were allowed to advance in the survey.

Informed consent acknowledgement

MFM fellowship program year

1st year fellow

2nd year fellow

3rd year fellow

Please select which race(s) best match your identity

American Indian and Alaska Native non-Hispanic

Asian non-Hispanic

Black or African American non-Hispanic

Hawaiian or Other Pacific Islander non-Hispanic

White non-Hispanic

Hispanic or Latina/o

Other: ____

Prefer not to disclose

What is your preferred gender identity?

Woman

Man

Transgender

Non-binary

I identify as: ___

Prefer not to say

Where is your fellowship located?

Northeast: Connecticut, Maine, Massachusetts, New Hampshire, New Jersey, New York, Pennsylvania, Rhode Island, Vermont

Midwest: Illinois, Indiana, Iowa, Kansas, Michigan, Minnesota, Missouri, Nebraska, North Dakota, Ohio, South Dakota, Wisconsin

Midwest: Illinois, Indiana, Iowa, Kansas, Michigan, Minnesota, Missouri, Nebraska, North Dakota, Ohio, South Dakota, Wisconsin

South: Alabama, Arkansas, Delaware, District of Columbia, Florida, Georgia, Kentucky, Louisiana, Maryland, Mississippi, North Carolina, Oklahoma, South Carolina, Tennessee, Texas, West Virginia

West: Alaska, Arizona, California, Colorado, Hawaii, Idaho, Montana, Nevada, New Mexico, Oregon, Utah, Washington, Wyoming

The following questions will ask about your experience with point-of-care ultrasound (POCUS). POCUS is a noninvasive, bedside extension to the physical exam which facilitates immediate assessment of conditions which benefit from timely diagnosis. For the purposes of this survey, maternal POCUS focuses on maternal assessment (e.g. fluid status, cardiac strain, intraabdominal hemorrhage) and does not include fetoplacental assessment.

Acknowledge

Have you *observed* maternal POCUS assessments on *pregnant* patients during fellowship?

Yes

No

How often do you yourself *perform* maternal POCUS to inform clinical decision-making?

Have not performed

< 1 time per month

1-3 times per month

≥ 1 time per week

≥ 1 time per day

Have you been trained to perform maternal POCUS exams?

Yes

No

If “Yes” to 9: Did maternal POCUS training occur during MFM fellowship?

Yes

No - Please specify training type and duration of training

If “Yes” to 9: Which training strategies did you use to learn maternal POCUS? Select all that apply.

Lectures

Online modules

Independent scanning

Guided scanning with instructor

Simulated session – mannequin

POCUS conference/workshop

Other - Please specify: ___

If “Yes” to 9: Please rank the effectiveness of the following educational strategies for maternal POCUS on a scale of 1 (very effective) to 5 (very ineffective)

Lectures

Online Modules

Independent scanning

Guided scanning with instructor

Simulated patient mannequins

POCUS conference/workshop

If “Yes” to 9: Which faculty or colleagues contributed to your maternal POCUS training? Select all that apply.

MFM Attending

MFM/Critical Care Attending

MFM Fellow

OBGYN Generalist Attending

Intensive Care Unit (ICU) Attending (non-MFM provider

ICU Fellow

Emergency Room Attending

Emergency Room Resident

Other - Please specify: ___

Do you have dedicated fellowship training in an Obstetric Intensive Care Unit?

Yes

No

How do you rate the importance of the following POCUS topics in MFM training? 1 = very important to 5 = very unimportant

Abdominal FAST (Focused Assessment with Sonography for Trauma)

Cardiac assessment

Inferior Vena Cava assessment

Lung assessment

Other: ___

Please mark your level of agreement with the following statement: I received adequate instruction to independently perform maternal POCUS during MFM fellowship.

Strongly Agree

Somewhat Agree

Neither agree or disagree

Somewhat disagree

Strongly disagree

Please mark your level of agreement with the following statement: I am interested in obtaining maternal POCUS training.

Strongly Agree

Somewhat Agree

Neither agree or disagree

Somewhat disagree

Strongly disagree

Please mark your level of agreement with the following statement: Training in maternal POCUS would enhance my clinical abilities.

Strongly Agree

Somewhat Agree

Neither agree or disagree

Somewhat disagree

Strongly disagree

I have interest in participating in the following to learn maternal POCUS:

Hands-on postgraduate course at SMFM or ISUOG

SMFM Fellow lecture or other online module

Not interested

Thank you for completing this survey. Would you like to participate in a drawing to win one of five $20 Amazon gift cards by providing an email address? Your email address will not be associated with your survey responses

Yes

No

Table 1 Fellow Program Regions Defined

Northeast: Connecticut, Maine, Massachusetts, New Hampshire, New Jersey, New York, Pennsylvania, Rhode Island, Vermont

Midwest: Illinois, Indiana, Iowa, Kansas, Michigan, Minnesota, Missouri, Nebraska, North Dakota, Ohio, South Dakota, Wisconsin

South: Alabama, Arkansas, Delaware, District of Columbia, Florida, Georgia, Kentucky, Louisiana, Maryland, Mississippi, North Carolina, Oklahoma, South Carolina, Tennessee, Texas, Virginia, West Virginia

West: Alaska, Arizona, California, Colorado, Hawaii, Idaho, Montana, Nevada, New Mexico, Oregon, Utah, Washington, Wyoming
